# 1,4,8,11-Tetra­kis(carboxy­meth­yl)-5,5,7,12,12,14-hexa­methyl-4,11-diaza-1,8-diazo­niacyclo­tetra­decane dichloride dihydrate

**DOI:** 10.1107/S1600536808010532

**Published:** 2008-04-23

**Authors:** Shi-Fan Wang, Hai-Qing Liu, Xue-Mei Yao, Kai Yang, Xiao-Hong Li

**Affiliations:** a Key Laboratory of Tropical Biological Resources of the Ministry of Education, Hainan University, Haikou 570228, People’s Republic of China, and Hainan Provincial Key Laboratory for Tropical Hydrobiology and Biotechnology, School of Ocean, Hainan University, Haikou 570228, People’s Republic of China; b Department of Pharmaceutical Engineering, School of Ocean, Hainan University, Haikou 570228, People’s Republic of China

## Abstract

The title compound, C_24_H_46_N_4_O_8_^2+^·2Cl^−^·2H_2_O, was synthes­ized by the hydrolysis of tetra­ethyl 2,2′,2′′,2′′′-(5,5,7,12,12,14-hexa­methyl-1,4,8,11-tetra­azacyclo­tetra­decane-1,4,8,11-tetra­yl) tetra­acetate in hydro­chloric acid solution. The crystal structure of the title compound consists of a 14-membered C_10_N_4_ centrosymmetric cationic macrocycle which inter­acts with the chloride ions and water mol­ecules of crystallization to give a three-dimensional hydrogen-bonded network.

## Related literature

For related literature, see: Marinelli *et al.* (2002 ▶); Wang (2001 ▶); Xu *et al.* (1988 ▶).
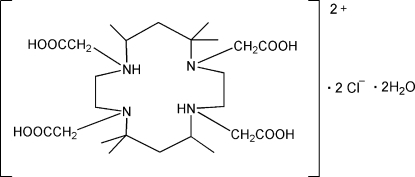


## Experimental

### 

#### Crystal data

C_24_H_46_N_4_O_8_^2+^·2Cl^−^·2H_2_O
*M* *_r_* = 625.58
Monoclinic, 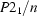

*a* = 9.977 (5) Å
*b* = 13.475 (7) Å
*c* = 11.572 (6) Å
β = 104.220 (9)°
*V* = 1508.1 (13) Å^3^
*Z* = 2
Mo *K*α radiation
μ = 0.27 mm^−1^
*T* = 293 (2) K
0.10 × 0.10 × 0.08 mm

#### Data collection

Bruker SMART CCD area-detector diffractometer
Absorption correction: none
15242 measured reflections
2946 independent reflections
2036 reflections with *I* > 2σ(*I*)
*R*_int_ = 0.143

#### Refinement

*R*[*F* ^2^ > 2σ(*F* ^2^)] = 0.056
*wR*(*F* ^2^) = 0.141
*S* = 0.98
2946 reflections
194 parameters
H atoms treated by a mixture of independent and constrained refinement
Δρ_max_ = 0.63 e Å^−3^
Δρ_min_ = −0.37 e Å^−3^

### 

Data collection: *SMART* (Bruker, 2001 ▶); cell refinement: *SAINT* (Bruker, 2001 ▶); data reduction: *SAINT*; program(s) used to solve structure: *SHELXTL* (Sheldrick, 2008 ▶); program(s) used to refine structure: *SHELXTL*; molecular graphics: *SHELXTL*; software used to prepare material for publication: *SHELXTL* and local programs.

## Supplementary Material

Crystal structure: contains datablocks global, I. DOI: 10.1107/S1600536808010532/wn2251sup1.cif

Structure factors: contains datablocks I. DOI: 10.1107/S1600536808010532/wn2251Isup2.hkl

Additional supplementary materials:  crystallographic information; 3D view; checkCIF report

## Figures and Tables

**Table 1:** Hydrogen-bond geometry (Å, °)

*D*—H⋯*A*	*D*—H	H⋯*A*	*D*⋯*A*	*D*—H⋯*A*
O4—H4⋯Cl1^i^	0.82	2.24	3.012 (3)	158
O2—H2*A*⋯O1*W*^ii^	0.82	1.82	2.610 (3)	162
O1*W*—H2*W*⋯Cl1^iii^	0.76 (3)	2.40 (3)	3.152 (3)	169 (3)

Symmetry codes: (i) 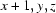; (ii) 
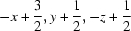
; (iii) 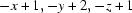.

**Fig. 1.**
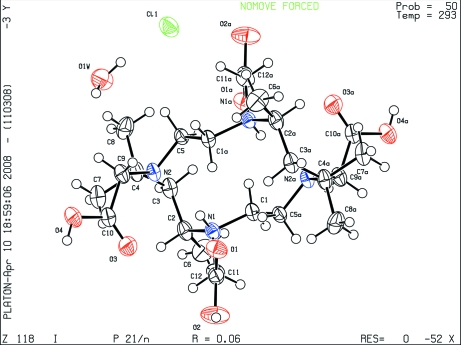
The structure of the title compound, showing 50% probability displacement ellipsoids and the atom-numbering scheme. Hydrogen atoms are drawn as spheres of arbitrary radius. Symmetry code: (a) 1-x, 2-y, -z.

**Fig. 2.**
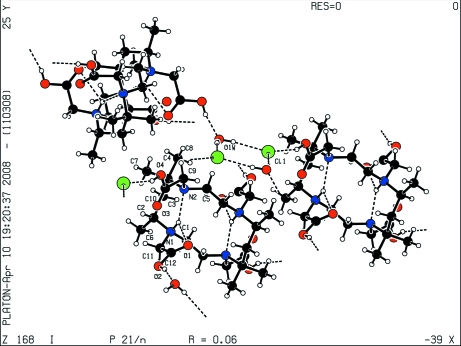
A view of the hydrogen bonding. Dashed lines indicate hydrogen bonds.

## supplementary crystallographic information

### Comment

N-functionalized macrocyclic acids are an important class of compounds for their utility as MRI contrast agents (Marinelli *et al.*, 2002) and their strong chelating ability (Xu *et al.*, 1988). For this reason, we have synthesized the title compound by the hydrolysis of tetraethyl 2,2',2'',2'''-(5,5,7,12,12,14-hexamethyl- 1,4,8,11-tetraazacyclotetradecane-1,4,8,11-tetrayl) tetraacetate (Wang *et al.*, 2001) in hydrochloric acid solution.

The bond lengths and angles in the title compound are within normal ranges. The structural data confirm that the 14-membered macrocycle lies on a center of inversion and each N atom is linked to a carboxymethyl group. The macrocycle carries two positive charges arising from the protonation of N atoms. The net charge is balanced by two chloride ions. The cations and anions interact with each other and with the water molecules of crystallization to furnish a hydrogen-bonded network structure (Table 1). The structure of the title compound, showing 50% probability displacement ellipsoids is shown in Fig. 1 and a view of the hydrogen bonding in Fig. 2.

### Experimental

Tetraethyl 2,2',2'',2'''-(5,5,7,12,12,14-hexamethyl- 1,4,8,11-tetraazacyclotetradecane-1,4,8,11-tetrayl)tetraacetate (0.625 g, 1 mmol) was dissolved in 200 ml of hydrochloric acid solution (v:v, 1:1) and allowed to stand in air at room temperature over a period of three weeks. Colourless block crystals suitable for X-ray diffraction analysis were formed at the bottom of the vessel (yield 87%).

### Refinement

The water hydrogen atoms were refined freely, resulting in O—H bond lengths of 0.76 (3) and 0.88 (5) Å. Other H atoms were positioned geometrically, with N—H = 0.91 Å, O—H = 0.82 Å, C—H = 0.96 Å for methyl, 0.97 Å for methylene and 0.98 Å for methine. U_iso_(H) = xU_eq_(carrier atom), where x = 1.5 for O and methyl, x = 1.2 for all other carrier atoms. The value of R_int_ (0.14) is high because of the quality of the diffraction data.

### Crystal data

**Table:**

C_24_H_46_N_4_O_8_^2+^·2Cl^–^·2H_2_O	*F*_000_ = 672
*M**_r_* = 625.58	*D*_x_ = 1.378 Mg m^−^^3^
Monoclinic, *P*2_1_/*n*	Mo *K*α radiation λ = 0.71073 Å
Hall symbol: -P 2yn	Cell parameters from 17651 reflections
*a* = 9.977 (5) Å	θ = 2.4–24.9º
*b* = 13.475 (7) Å	µ = 0.27 mm^−^^1^
*c* = 11.572 (6) Å	*T* = 293 (2) K
β = 104.220 (9)º	BLOCK, colorless
*V* = 1508.1 (13) Å^3^	0.10 × 0.10 × 0.08 mm
*Z* = 2	

### Data collection

**Table:**

Bruker SMART CCD area-detector diffractometer	2036 reflections with *I* > 2σ(*I*)
Radiation source: fine-focus sealed tube	*R*_int_ = 0.143
Monochromator: graphite	θ_max_ = 26.0º
*T* = 293(2) K	θ_min_ = 2.4º
φ and ω scans	*h* = −12→12
Absorption correction: none	*k* = −16→16
15242 measured reflections	*l* = −14→14
2946 independent reflections	

### Refinement

**Table:**

Refinement on *F*^2^	Secondary atom site location: difference Fourier map
Least-squares matrix: full	Hydrogen site location: inferred from neighbouring sites
*R*[*F*^2^ > 2σ(*F*^2^)] = 0.056	H atoms treated by a mixture of independent and constrained refinement
*wR*(*F*^2^) = 0.141	*w* = 1/[σ^2^(*F*_o_^2^) + (0.0715*P*)^2^] where *P* = (*F*_o_^2^ + 2*F*_c_^2^)/3
*S* = 0.99	(Δ/σ)_max_ = 0.032
2946 reflections	Δρ_max_ = 0.63 e Å^−^^3^
194 parameters	Δρ_min_ = −0.37 e Å^−^^3^
Primary atom site location: structure-invariant direct methods	Extinction correction: none

### Special details

**Table:**

Geometry. All e.s.d.'s (except the e.s.d. in the dihedral angle between two l.s. planes) are estimated using the full covariance matrix. The cell e.s.d.'s are taken into account individually in the estimation of e.s.d.'s in distances, angles and torsion angles; correlations between e.s.d.'s in cell parameters are only used when they are defined by crystal symmetry. An approximate (isotropic) treatment of cell e.s.d.'s is used for estimating e.s.d.'s involving l.s. planes.
Refinement. Refinement of *F*^2^ against ALL reflections. The weighted *R*-factor *wR* and goodness of fit *S* are based on *F*^2^, conventional *R*-factors *R* are based on *F*, with *F* set to zero for negative *F*^2^. The threshold expression of *F*^2^ > σ(*F*^2^) is used only for calculating *R*-factors(gt) *etc*. and is not relevant to the choice of reflections for refinement. *R*-factors based on *F*^2^ are statistically about twice as large as those based on *F*, and *R*- factors based on ALL data will be even larger.

### Fractional atomic coordinates and isotropic or equivalent isotropic displacement parameters (Å^2^)

**Table:**

	*x*	*y*	*z*	*U*_iso_*/*U*_eq_	
C1	0.5351 (3)	0.98193 (17)	−0.1532 (2)	0.0292 (6)	
H1A	0.4667	0.9400	−0.1306	0.035*	
H1B	0.5360	0.9664	−0.2348	0.035*	
C2	0.7189 (3)	0.85672 (17)	−0.0818 (2)	0.0335 (6)	
H2	0.8150	0.8530	−0.0349	0.040*	
C3	0.6370 (3)	0.78525 (17)	−0.0239 (2)	0.0323 (6)	
H3A	0.6485	0.7189	−0.0525	0.039*	
H3B	0.5398	0.8021	−0.0511	0.039*	
C4	0.6741 (3)	0.78252 (17)	0.1120 (2)	0.0319 (6)	
C5	0.5034 (2)	0.90943 (16)	0.1443 (2)	0.0285 (6)	
H5A	0.4752	0.8726	0.2064	0.034*	
H5B	0.4516	0.8836	0.0682	0.034*	
C6	0.7192 (4)	0.8235 (2)	−0.2076 (3)	0.0495 (8)	
H6A	0.6257	0.8156	−0.2537	0.074*	
H6B	0.7673	0.7614	−0.2041	0.074*	
H6C	0.7649	0.8726	−0.2444	0.074*	
C7	0.8226 (3)	0.7482 (2)	0.1616 (3)	0.0422 (7)	
H7A	0.8371	0.6866	0.1248	0.063*	
H7B	0.8394	0.7391	0.2462	0.063*	
H7C	0.8850	0.7974	0.1450	0.063*	
C8	0.5799 (3)	0.70891 (19)	0.1556 (3)	0.0442 (7)	
H8A	0.6022	0.6425	0.1369	0.066*	
H8B	0.4851	0.7228	0.1169	0.066*	
H8C	0.5933	0.7154	0.2403	0.066*	
C9	0.7378 (2)	0.91341 (19)	0.2780 (2)	0.0328 (6)	
H9A	0.7422	0.8550	0.3278	0.039*	
H9B	0.6935	0.9660	0.3121	0.039*	
C10	0.8828 (3)	0.94509 (17)	0.2765 (2)	0.0303 (6)	
C11	0.7806 (3)	1.03008 (17)	−0.0922 (2)	0.0338 (6)	
H11A	0.7562	1.0512	−0.1749	0.041*	
H11B	0.8669	0.9937	−0.0793	0.041*	
C12	0.8052 (3)	1.12186 (18)	−0.0142 (2)	0.0319 (6)	
Cl1	0.27157 (7)	0.97170 (5)	0.43440 (7)	0.0480 (3)	
N1	0.6734 (2)	0.96203 (13)	−0.07355 (18)	0.0270 (5)	
H1N	0.6660	0.9714	0.0025	0.032*	
N2	0.6540 (2)	0.89074 (14)	0.15548 (17)	0.0275 (5)	
O1	0.74179 (19)	1.14840 (14)	0.05549 (17)	0.0405 (5)	
O2	0.9133 (2)	1.17039 (15)	−0.0337 (2)	0.0572 (6)	
H2A	0.9260	1.2212	0.0065	0.086*	
O3	0.9143 (2)	0.97572 (14)	0.19069 (18)	0.0449 (5)	
O4	0.96439 (19)	0.93712 (15)	0.38441 (16)	0.0434 (5)	
H4	1.0409	0.9596	0.3849	0.065*	
O1W	0.5390 (3)	0.84981 (16)	0.4486 (2)	0.0519 (6)	
H2W	0.594 (4)	0.887 (2)	0.479 (3)	0.042 (10)*	
H1W	0.457 (5)	0.877 (3)	0.443 (4)	0.092 (14)*	

### Atomic displacement parameters (Å^2^)

**Table:**

	*U*^11^	*U*^22^	*U*^33^	*U*^12^	*U*^13^	*U*^23^
C1	0.0262 (13)	0.0260 (12)	0.0318 (13)	0.0029 (10)	0.0005 (11)	0.0006 (10)
C2	0.0304 (14)	0.0251 (12)	0.0417 (15)	0.0084 (11)	0.0026 (12)	−0.0014 (11)
C3	0.0336 (14)	0.0220 (12)	0.0357 (14)	0.0030 (11)	−0.0019 (11)	−0.0050 (10)
C4	0.0332 (14)	0.0217 (12)	0.0356 (14)	0.0055 (10)	−0.0012 (11)	−0.0029 (10)
C5	0.0218 (12)	0.0263 (12)	0.0340 (14)	−0.0011 (10)	0.0002 (11)	0.0031 (10)
C6	0.070 (2)	0.0378 (15)	0.0471 (18)	0.0086 (15)	0.0258 (16)	−0.0046 (13)
C7	0.0435 (17)	0.0368 (14)	0.0398 (15)	0.0101 (13)	−0.0021 (13)	−0.0001 (12)
C8	0.0569 (19)	0.0261 (13)	0.0446 (16)	−0.0013 (13)	0.0031 (14)	0.0076 (12)
C9	0.0271 (13)	0.0387 (14)	0.0279 (13)	0.0018 (11)	−0.0022 (11)	−0.0041 (11)
C10	0.0298 (14)	0.0239 (12)	0.0324 (14)	0.0020 (10)	−0.0017 (11)	−0.0037 (10)
C11	0.0292 (13)	0.0320 (13)	0.0405 (15)	−0.0007 (11)	0.0094 (12)	−0.0045 (11)
C12	0.0279 (14)	0.0287 (13)	0.0365 (14)	−0.0008 (11)	0.0029 (12)	0.0030 (11)
Cl1	0.0331 (4)	0.0426 (4)	0.0634 (5)	−0.0038 (3)	0.0023 (3)	0.0034 (3)
N1	0.0249 (11)	0.0246 (10)	0.0298 (11)	0.0013 (8)	0.0036 (9)	−0.0025 (8)
N2	0.0248 (11)	0.0229 (10)	0.0298 (11)	0.0027 (8)	−0.0025 (9)	−0.0029 (8)
O1	0.0330 (10)	0.0486 (11)	0.0384 (11)	−0.0090 (9)	0.0060 (9)	−0.0126 (9)
O2	0.0537 (14)	0.0430 (12)	0.0839 (17)	−0.0206 (10)	0.0339 (13)	−0.0177 (11)
O3	0.0393 (12)	0.0501 (12)	0.0415 (12)	−0.0057 (9)	0.0023 (9)	0.0060 (9)
O4	0.0286 (10)	0.0547 (12)	0.0392 (11)	−0.0042 (9)	−0.0065 (9)	−0.0003 (9)
O1W	0.0484 (15)	0.0327 (11)	0.0759 (17)	0.0025 (11)	0.0176 (13)	0.0018 (11)

### Geometric parameters (Å, °)

**Table:**

C1—N1	1.483 (3)	C7—H7B	0.9600
C1—C5^i^	1.523 (3)	C7—H7C	0.9600
C1—H1A	0.9700	C8—H8A	0.9600
C1—H1B	0.9700	C8—H8B	0.9600
C2—N1	1.500 (3)	C8—H8C	0.9600
C2—C3	1.521 (4)	C9—N2	1.490 (3)
C2—C6	1.524 (4)	C9—C10	1.513 (4)
C2—H2	0.9800	C9—H9A	0.9700
C3—C4	1.525 (4)	C9—H9B	0.9700
C3—H3A	0.9700	C10—O3	1.186 (3)
C3—H3B	0.9700	C10—O4	1.317 (3)
C4—C7	1.523 (4)	C11—N1	1.465 (3)
C4—C8	1.535 (4)	C11—C12	1.515 (4)
C4—N2	1.571 (3)	C11—H11A	0.9700
C5—N2	1.497 (3)	C11—H11B	0.9700
C5—C1^i^	1.523 (3)	C12—O1	1.196 (3)
C5—H5A	0.9700	C12—O2	1.327 (3)
C5—H5B	0.9700	N1—H1N	0.9100
C6—H6A	0.9600	O2—H2A	0.8200
C6—H6B	0.9600	O4—H4	0.8200
C6—H6C	0.9600	O1W—H2W	0.76 (3)
C7—H7A	0.9600	O1W—H1W	0.88 (5)
			
N1—C1—C5^i^	110.10 (18)	C4—C7—H7C	109.5
N1—C1—H1A	109.6	H7A—C7—H7C	109.5
C5^i^—C1—H1A	109.6	H7B—C7—H7C	109.5
N1—C1—H1B	109.6	C4—C8—H8A	109.5
C5^i^—C1—H1B	109.6	C4—C8—H8B	109.5
H1A—C1—H1B	108.2	H8A—C8—H8B	109.5
N1—C2—C3	111.5 (2)	C4—C8—H8C	109.5
N1—C2—C6	114.2 (2)	H8A—C8—H8C	109.5
C3—C2—C6	111.3 (2)	H8B—C8—H8C	109.5
N1—C2—H2	106.4	N2—C9—C10	111.2 (2)
C3—C2—H2	106.4	N2—C9—H9A	109.4
C6—C2—H2	106.4	C10—C9—H9A	109.4
C2—C3—C4	116.7 (2)	N2—C9—H9B	109.4
C2—C3—H3A	108.1	C10—C9—H9B	109.4
C4—C3—H3A	108.1	H9A—C9—H9B	108.0
C2—C3—H3B	108.1	O3—C10—O4	126.4 (3)
C4—C3—H3B	108.1	O3—C10—C9	123.9 (2)
H3A—C3—H3B	107.3	O4—C10—C9	109.6 (2)
C7—C4—C3	111.3 (2)	N1—C11—C12	116.1 (2)
C7—C4—C8	107.3 (2)	N1—C11—H11A	108.3
C3—C4—C8	110.0 (2)	C12—C11—H11A	108.3
C7—C4—N2	110.47 (19)	N1—C11—H11B	108.3
C3—C4—N2	106.86 (18)	C12—C11—H11B	108.3
C8—C4—N2	111.0 (2)	H11A—C11—H11B	107.4
N2—C5—C1^i^	114.86 (19)	O1—C12—O2	123.7 (2)
N2—C5—H5A	108.6	O1—C12—C11	127.7 (2)
C1^i^—C5—H5A	108.6	O2—C12—C11	108.7 (2)
N2—C5—H5B	108.6	C11—N1—C1	113.39 (19)
C1^i^—C5—H5B	108.6	C11—N1—C2	109.8 (2)
H5A—C5—H5B	107.5	C1—N1—C2	112.43 (18)
C2—C6—H6A	109.5	C11—N1—H1N	106.9
C2—C6—H6B	109.5	C1—N1—H1N	106.9
H6A—C6—H6B	109.5	C2—N1—H1N	106.9
C2—C6—H6C	109.5	C9—N2—C5	111.37 (19)
H6A—C6—H6C	109.5	C9—N2—C4	114.14 (17)
H6B—C6—H6C	109.5	C5—N2—C4	109.45 (17)
C4—C7—H7A	109.5	C12—O2—H2A	109.5
C4—C7—H7B	109.5	C10—O4—H4	109.5
H7A—C7—H7B	109.5	H2W—O1W—H1W	107 (3)
			
N1—C2—C3—C4	−75.6 (3)	C6—C2—N1—C11	−71.7 (3)
C6—C2—C3—C4	155.6 (2)	C3—C2—N1—C1	−71.7 (3)
C2—C3—C4—C7	−62.6 (3)	C6—C2—N1—C1	55.5 (3)
C2—C3—C4—C8	178.6 (2)	C10—C9—N2—C5	−151.36 (19)
C2—C3—C4—N2	58.1 (3)	C10—C9—N2—C4	84.1 (2)
N2—C9—C10—O3	20.5 (3)	C1^i^—C5—N2—C9	67.6 (3)
N2—C9—C10—O4	−162.1 (2)	C1^i^—C5—N2—C4	−165.3 (2)
N1—C11—C12—O1	−5.1 (4)	C7—C4—N2—C9	−34.3 (3)
N1—C11—C12—O2	174.1 (2)	C3—C4—N2—C9	−155.5 (2)
C12—C11—N1—C1	92.6 (3)	C8—C4—N2—C9	84.6 (2)
C12—C11—N1—C2	−140.7 (2)	C7—C4—N2—C5	−159.9 (2)
C5^i^—C1—N1—C11	−52.7 (3)	C3—C4—N2—C5	78.9 (2)
C5^i^—C1—N1—C2	−178.0 (2)	C8—C4—N2—C5	−41.0 (2)
C3—C2—N1—C11	161.1 (2)		

Symmetry codes: (i) −*x*+1, −*y*+2, −*z*.

### Hydrogen-bond geometry (Å, °)

**Table:**

*D*—H···*A*	*D*—H	H···*A*	*D*···*A*	*D*—H···*A*
O4—H4···Cl1^ii^	0.82	2.24	3.012 (3)	158
O2—H2A···O1W^iii^	0.82	1.82	2.610 (3)	162
O1W—H2W···Cl1^iv^	0.76 (3)	2.40 (3)	3.152 (3)	169 (3)

Symmetry codes: (ii) *x*+1, *y*, *z*; (iii) −*x*+3/2, *y*+1/2, −*z*+1/2; (iv) −*x*+1, −*y*+2, −*z*+1.

## References

[bb1] Bruker (2001). *SAINT* and *SMART* Bruker AXS Inc., Madison, Wisconsin, USA.

[bb2] Marinelli, E. R., Neubeck, R., Song, B., Wagler, T., Ranganathan, R. S., Sukumaran, K., Wedeking, P., Nunn, A., Runge, V. & Tweedle, M. (2002). *Acad. Radiol.* **9**, s251–s254. 10.1016/s1076-6332(03)80449-x 12019882

[bb3] Sheldrick, G. M. (2008). *Acta Cryst.* A**64**, 112–122. 10.1107/S0108767307043930 18156677

[bb4] Wang, S. F. (2001). *Chin. J. Syn. Chem.* **9**, 223–226, 231.

[bb5] Xu, J. D., Ni, S. S. & Lin, Y. J. (1988). *Inorg. Chem. *, 4651–4657.

